# Gut microbiome and serum amino acid metabolome alterations in autism spectrum disorder

**DOI:** 10.1038/s41598-024-54717-2

**Published:** 2024-02-19

**Authors:** Xuening Chang, Yuchen Zhang, Xue Chen, Shihan Li, Hong Mei, Han Xiao, Xinyu Ma, Zhisheng Liu, Ruizhen Li

**Affiliations:** 1 Department of Child Health Care, Wuhan Children’s Hospital, Tongji Medical College, Huazhong University of Science and Technology, Wuhan, 430016 China; 2 School of Medicine, Wuhan University of Science and Technology, Wuhan, 430081 China; 3 Department of Maternal and Child Health, Wuhan Children’s Hospital, Tongji Medical College, Huazhong University of Science and Technology, Wuhan, 430016 China; 4 Department of Radiology, Wuhan Children’s Hospital, Tongji Medical College, Huazhong University of Science and Technology, Wuhan, 430016 China; 5 Wuhan Children’s Hospital, Tongji Medical College, Huazhong University of Science and Technology, Wuhan, 430016 China

**Keywords:** ASD, Gut microbiota, Metagenomics, Metabolism, Neurological disorders, Paediatrics

## Abstract

Gut microbiota and their metabolic products might play important roles in regulating the pathogenesis of autism spectrum disorder (ASD). The purpose of this study was to characterize gut microbiota and serum amino acid metabolome profiles in children with ASD. A non-randomized controlled study was carried out to analyze the alterations in the intestinal microbiota and their metabolites in patients with ASD (n = 30) compared with neurotypical controls (NC) (n = 30) by metagenomic sequencing to define the gut microbiota community and liquid chromatography/mass spectrometry (LC/MS) analysis to characterize the metabolite profiles. Compared with children in the NC group, those in the ASD group showed lower richness, higher evenness, and an altered microbial community structure. At the class level, *Deinococci* and *Holophagae* were significantly lower in children with ASD compared with TD. At the phylum level, *Deinococcus*-Thermus was significantly lower in children with ASD compared with TD. In addition, the functional properties (such as galactose metabolism) displayed significant differences between the ASD and NC groups. Five dominant altered species were identified and analyzed (LDA score > 2.0, *P* < 0.05), including *Subdoligranulum*, *Faecalibacterium*_praushitzii, *Faecalibacterium*, *Veillonellaceae*, and *Rumminococcaceae*. The peptides/nickel transport system was the main metabolic pathway involved in the differential species in the ASD group. Decreased ornithine levels and elevated valine levels may increase the risk of ASD through a metabolic pathway known as the nickel transport system. The microbial metabolism in diverse environments was negatively correlated with *phascolarctobacterium succinatutens*. Our study provides novel insights into compositional and functional alterations in the gut microbiome and metabolite profiles in ASD and the underlying mechanisms between metabolite and ASD.

## Introduction

Autism spectrum disorder (ASD) is a neurodevelopmental disorder characterized by deficits in social communication, narrow and restricted interests, and repetitive behavior, which usually begin in early childhood^1^. The incidence of ASD has increased dramatically in recent years^2^. The prevalence of ASD has increased from 1 in 44 in 2021 to 1 in 36 according to 2023 data from the United States Centers for Disease Control and Prevention^3,4^. Coupled with high disability rates and poor outcomes, ASD imposes a heavy burden on society. However, the cause is unknown and the core symptoms can only be improved through rehabilitation training. Therefore, it is urgent to find out the pathogenesis of ASD and further find effective treatment methods.

Etiological studies have shown that both genetic and environmental factors influence the onset of neurological diseases. Environmental factors include exposure to toxins, diet and nutrition, and infections^5^. Among them, intestinal microorganisms can affect central nervous function through immune, neuroendocrine, neurotransmitter, metabolism, and other pathways through the "gut-brain axis"^6^. The gut-brain axis is the information exchange system between the gut and the brain, which is composed of immune, vagus, and neuroendocrine pathways^7^. In the process of intestinal metabolism, intestinal flora produces 5-hydroxytryptamine, catecholamine, gamma-aminobutyric acid, and other neurotransmitters and short-chain fatty acids and amino acid metabolites, which can activate the afferent nerve of the intestinal nervous system and affect the central nervous system through the surface receptors of intestinal epithelial cells and intestinal pheochromocytomes^8,9^.

There is increasing evidence that intestinal flora and metabolites influence the disease course of ASD. Studies have shown that children with ASD often have gastrointestinal symptoms such as constipation, diarrhea, and abdominal pain, which can aggravate behavioral problems such as rigidity, hyperactivity, and social withdrawal^10,11^. Animal experiments^12^ confirmed that fecal transplantation of ASD patients into the intestines of sterile mice showed behavioral defects in the offspring of the mice, showing more repetitive behaviors and decreased social and motor ability compared with the control group^13^. This suggests that the intestinal flora of patients with ASD may induce abnormal behavior. Although many studies have shown that changes in intestinal flora in patients with ASD play an important role in the development of the disease, the role of metabolites has been less considered^14^. Intestinal metabolites also play an important role in the development of ASD. For example, most studies^15^ show that propionic acid concentration in the feces of children with ASD is significantly higher; Butyric acid increases social behavior in mice by modulating the excitatory/inhibitory system^16^.

Based on these results, remodeling intestinal flora has attracted increasing attention in the treatment of ASD. Kang et al. treated ASD children with microbial transfer therapy, and the gastrointestinal and behavioral symptoms of the children were significantly improved 8 weeks after treatment^17^, and the symptoms still improved two years after treatment^18^. However, due to individual differences in children with ASD and different research methods, there is no unified understanding of the different strains, but often contradictory. To date, neither a specific microbiome composition nor a pathogenic strain causing ASD has been identified^19^. Therefore, although gut flora plays a role in the treatment of ASD, specific changes in gut microbiota in children with ASD have not been verified.

In conclusion, the impact of ASD is becoming more and more serious. The sooner the pathogenesis of ASD is understood and effective treatment is found, the sooner children, families, and society will benefit. All these require us to carry out in-depth and detailed research on ASD. In the current study, we explored the relationship between gut microbiota and ASD, a correlation analysis between serum metabolites and metagenomics was also conducted.

## Subjects and methods

### Participants

Between January 2021 and June 2021, 30 children with ASD between 2 and 4 years of age (20 males and 10 females) were recruited from the Child Health Department of Wuhan Children's Hospital, China, while the 30 sex- and age-matched healthy children, who were not related to the autistic individuals, were recruited from the physical examination center of Wuhan Children's Hospital. All the children underwent neurological, physical, and behavioral examinations. Participants’ metadata was obtained about the age, sex, birth length, birth weight, type of delivery, feeding, dietary habits, paternal educational level, maternal educational level and habitation, and illness and medication was collected at the time of sampling. We classified the parent educational level into four categories: primary or less, secondary, university, or postgraduate or above.

Children with ASD were excluded from the study if they had a history of congenital diseases, and acute or chronic affective diseases in the past 3 months. All participants had not taken antibiotics, probiotics, prebiotics, or other medications that could influence the intestinal microbiota in the 3 months before the feces collection.

Written informed consent was obtained from the parents of all participants. All procedures were performed according to the principles of the Declaration of Helsinki and were approved by the ethics committee of the Wuhan Children's Hospital (Ethics NO, 2020R112-E02).

### Diagnosis and clinical measures

The children with ASD in this study were diagnosed according to the Diagnostic and Statistical Manual of Mental Disorders, 5th Edition (DSM-5). Dietary habits were evaluated according to the type of diet by interviewing the parents with a questionnaire^20^, including dairy products, rice or noodles, meat or eggs, bean products, vegetables and fruits, and other snacks. Individuals with five or more diet types were considered the normal group, while those with less than five types were considered the single group. The diagnosis was confirmed by two experienced developmental and behavioral pediatricians using the Childhood Autism Rating Scale (CARS).

### Fecal and blood sample collection

Feces were collected according to the instructions and delivered immediately at low temperatures during the day. Once received, one gram of stool was preserved in sterile storage tubes and stored at − 80 °C until DNA extraction. Blood samples (about 2–3 ml) were injected into procoagulant and anticoagulant tubes. Tubes were then sent to the laboratory for centrifugation and stored at − 80 °C for amino acid measurements.

### DNA extraction, library preparation, and metagenomic sequencing

The microbial community DNA was extracted using MagPure Stool DNA KF kit B (Magen, China) following the manufacturer's instructions. DNA was quantified with a Qubit Fluorometer by using a Qubit dsDNA BR Assay kit (Invitrogen, USA), and the quality was checked by running an aliquot on 1% agarose gel^21^. 1 μg genomic DNA was randomly fragmented by Covaris. The fragmented DNA was selected by Magnetic beads to an average size of 200-400 bp. The PCR primers used for library preparation were: Read1 (GA ACGACATGGCTACGA) and Read2 (TGTGAGCCAAGGAGTTG). The selected fragments were through end-repair, 3’ adenylated, adapters-ligation, and PCR Amplifying, and the products were purified by the Magnetic beads. The double-stranded PCR products were heat-denatured and circularized by the splint oligo sequence. The single-strand circle DNA (ssCir DNA) was formatted as the final library and qualified by QC^22^. The qualified libraries were sequenced on the MGISEQ-2000 platform (BGI Shenzhen, China).

### Bioinformatics analysis

The raw sequencing data were analyzed using a bioinformatics pipeline developed by BGI. The procedure briefly includes the following steps: (1) excluding Reads containing 10% uncertain bases (N bases); (2) excluding Reads containing adapter sequences (15 bases or longer sequence aligned to the adapter sequence); (3) excluding Reads containing low-quality bases of 20% (bases of Q < 20); and (4) for the sample with the host or environment sources, a filtering step is added here to remove the sequence of the host genome to reduce the interference of the host sequence on the subsequent analysis^23^. High-quality short reads of each DNA sample were assembled by the MEGAHIT^24^. Sequences are sorted in order of decreasing length. The longest sequence becomes the representative of the first cluster. Then, each remaining sequence is compared with the representatives of existing clusters. If the similarity with any representative is above 95%, it is grouped into that cluster. Otherwise, a new cluster is defined with that sequence as the representative^25^.

### Amino acid measurements

40 μl plasma was deproteinized with 20 μl 10% (w/v) sulfosalicylic acid (Sigma) containing internal standards, then 120 μl aqueous solution was added. After centrifuging, the supernatant was used for LC/MS analysis. The LC/MS analysis was performed by ultra-high pressure liquid chromatography (UHPLC) coupled to an AB Sciex Qtrap 5500 mass spectrometry (AB Sciex, US) with the electrospray ionization (ESI) source in positive ion mode. A Waters ACQUITY UPLC HSS T3 column (1.8 μm, 2.1 × 100 mm) was used for amino compound separation with a flow rate of 0.5 ml/min and column temperature of 55 °C. The mobile phases were (A) water containing 0.05% and 0.1% formic acid (v/v), and (B) acetonitrile containing 0.05% and 0.1% formic acid (v/v). The gradient elution was 2% B kept for 0.5 min, then changed linearly to 10% B for 1 min, continued up to 35% B in 2 min, increased to 95% B in 0.1 min, and maintained for 1.4 min. Multiple Reaction Monitoring (MRM) was used to monitor all amino compounds. The mass parameters were as follows, Curtain gas flow was 35 L/min, Collision Gas (CAD) was medium, Ion Source Gas 1 (GS 1) flow was 60 l/min, Ion Source Gas 2 (GS 2) flow was 60 l/min, IonSpray Voltage (IS) 5500 V, temperature 600 °C. All amino compound standards were purchased from Sigma and Toronto Research Chemical (TRC)^26^.

### Statistical analyses

Continuous variables were presented as mean ± standard deviation, and comparisons between ASD and typically developing (TD) groups were performed with paired Student *t*-test or Wilcoxon matched pairs test. Categorical variables were presented as proportions, and the groups were compared using chi-square tests. The differences in the alpha diversity and beta diversity between the groups were tested by the Wilcoxon rank-sum test. The indicators of alpha diversity included the Shannon index, Chao1, and Simpson index. Beta diversity measures the differences between groups, which is studied by the calculation of JSD distance/Bray Curtis distance from the abundance matrix. To identify taxa differentially represented between any two groups, differentially abundant taxa were selected using the LEfSe (linear discriminant analysis (LDA)) effect size^27^. Differential abundance of class and phyla between any two groups was tested by the Wilcoxon rank-sum test, *P* value was corrected as false discovery rate (FDR) with the Benjamini–Hochberg method^28^. ReporterScore^29^ is applied to carry out the statistical tests for all KO in the KEGG pathway. Change of the KEGG path can therefore be studied by the accumulation of statistical test results. PLS-DA^30,31^ was used to evaluate the difference in metabolic profiles between TD and ASD children. The significant metabolites with variable important in projection (VIP) ≥ 1, and *P* value (T-test) < 0.05. Spearman’s correlations were used to evaluate the correlation between differential species and differential metabolites. Differential species were tested by fold change analysis, and species with ≥ 1.2-fold changes and *P* < 0.05 were used as screening conditions. Canonical correspondence analysis (CCA)^32^ was performed to explore which metabolites are closely associated with changes in differential species based on the results of Spearman’s correlations. ReporterScore was performed for KEGG Module enrichment analysis. Then Spearman’s correlations were carried out between significant enriched KEGG Module and differential metabolites.

### Ethics approval and consent to participant

All procedures performed in studies involving human participants were by the ethical standards of the institutional and/or national research committee and with the 1964 Helsinki Declaration and its later amendments or comparable ethical standards. The present study was approved by the medical ethical committee of Wuhan Children's Hospital, Tongji Medical College, Huazhong University of Science and Technology (NO. 2020R112). Informed and signed consent was obtained from all parent(s)/caregiver(s) to the participating children.

## Results

### Participants

A total of 30 subjects with a clinical diagnosis of ASD children (average age 3.44 ± 0.85; sex, male: female 20:10) were recruited. Meanwhile, 30 age and sex-matched TD individuals (average age 3.28 ± 0.65; sex, male: female 20:10) who attended annual physical examinations were also recruited. Table 1 shows the characteristics of the subjects. We observed two significant differences in the distribution of birth length and habitation between the two groups (*p* = 0.014, *p* = 0.003, respectively). No significant differences were found in the other characteristics between the ASD and TD groups.

**Table 1:** Characteristics of study participants.

Characteristic	ASD	TD	*P* value
Subjects (n)	30	30	–
Age, year	3.44 ± 0.85	3.28 ± 0.65	0.408
Sex (n, %)			
Male	20 (66.67%)	20 (66.67%)	1.000
Female	10 (33.33%)	10 (33.33%)	
Birth length, cm	49.87 ± 1.33	50.83 ± 1.60	0.014
Birth weight, kg	3.43 ± 0.75	3.42 ± 0.38	0.940
Gestational age (n, %)			
Term	28 (93.33%)	29 (96.67%)	0.554
Preterm	2 (6.67%)	1 (3.33%)	
Delivery mode (n, %)			
Cesarean section	17 (56.67%)	21 (70.00%)	0.284
Natural birth	13 (43.33%)	9 (30.00%)	
Dietary habits			
Normal	14 (46.67)	21 (70.00%)	0.067
Single	16 (57.33%)	9 (30.00%)	
Feeding patterns (n, %)			
Breastfeeding	18 (60.00%)	15 (50.00%)	0.689
Artificial feeding	4 (13.33%)	4 (13.33%)	
Mixed feeding	8 (26.67%)	11 (36.67%)	
Paternal educational level (n, %)			
Primary or less	0 (00.00%)	0 (00.00%)	4.000
Secondary	8 (26.67%)	4 (13.33%)	
University	21 (70.00%)	21 (70.00%)	
Postgraduate or above	1 (3.33%)	5 (16.67%)	
Maternal educational level (n, %)			
Primary or less	0 (00.00%)	0 (00.00%)	5.946
Secondary	9 (30.00%)	4 (13.33%)	
University	21 (70.00%)	22 (73.33%)	
Postgraduate or above	0 (00.00%)	4 (13.33%)	
Habitation			
Urban	20 (66.67%)	29 (96.67%)	0.003
Rural	10 (33.33%)	1 (3.33%)	
CARS test	31.97 ± 2.94	NA	–

*ASD* autism spectrum disorders, *TD* typically developing.

### Metagenomic sequencing revealed significant differences between the ASD group and the TD group

**Figure 1:**
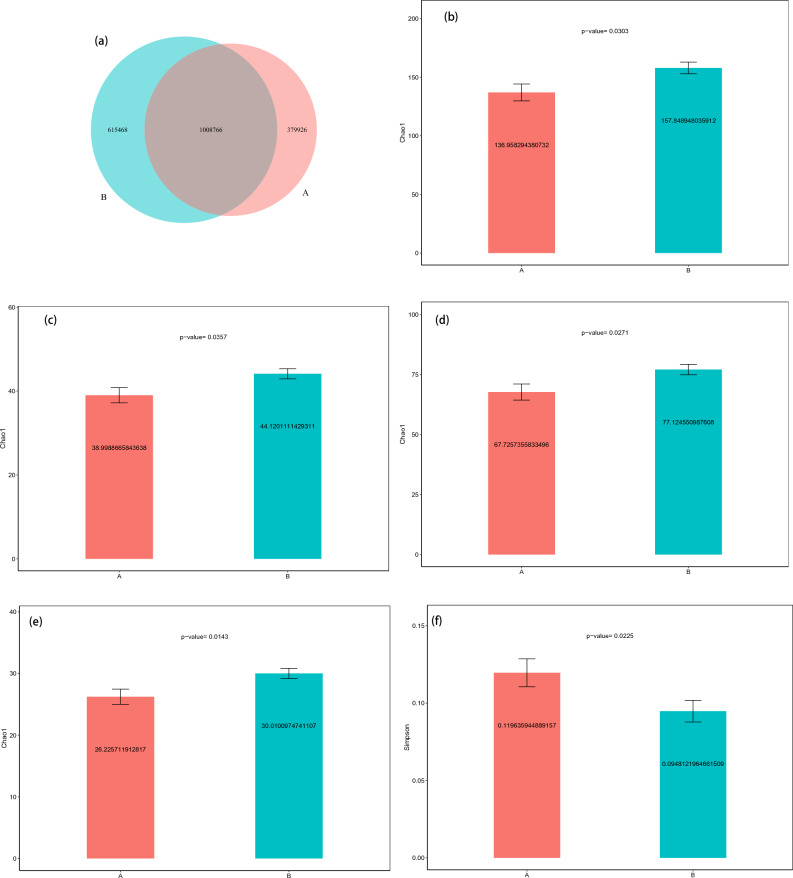

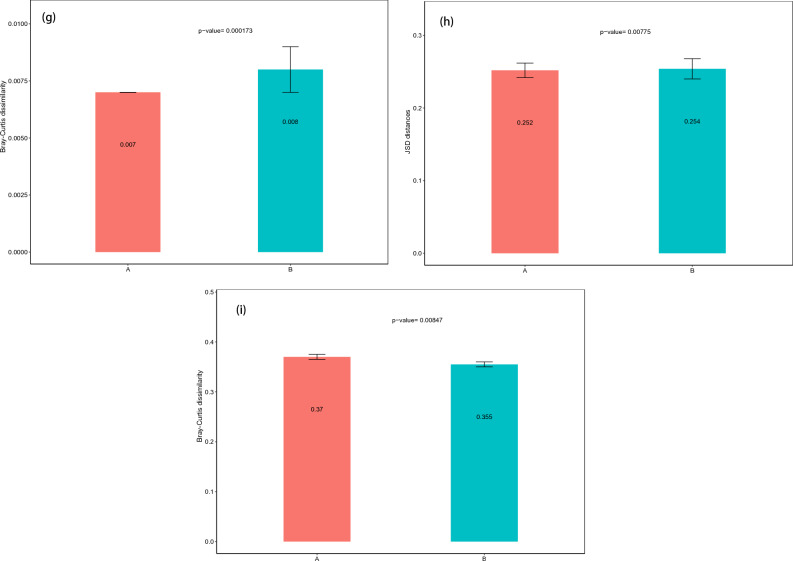
Differences in microbiota between ASD and neurotypical control groups. (**a**) Venn diagram of the observed overlapping genes in TD and ASD. (**b**) The estimate of Chao 1 index analysis between the TD (green-colored) and ASD (red-colored) groups (*P* < 0.05, Wilcoxon rank-sum test) at the level of family (**b**), class (**c**), order (**d**), and phylum (**e**). The estimate of Simpson index analysis between two groups at the level of kingdom (**f**). Beta diversity indices of kingdom for TD and ASD (*P* < 0.05, Wilcoxon rank-sum test). Estimate of Bray–Curtis dissimilarity (**g**) and JSD distances (**h**). Beta diversity indices of species for TD and ASD (*P* < 0.05, Wilcoxon rank-sum test). Estimate of Bray–Curtis dissimilarity (**i**). *ASD* autism spectrum disorder, *TD* typically developing.

As shown in Fig. 1a, the samples from the TD (B) group contained 615,468 specific genes, however, only 339,926 specific genes were found in the samples from the ASD (A) group. Species richness was significantly lower in the ASD group than in the TD group, as measured by Chao1 index analyses (Fig. 1b–e). However, the evenness of species at the kingdom level was significantly higher in the ASD group than in the TD group, as measured by Simpson index analyses (Fig. 1f). There were significant differences in beta diversity between the ASD group and TD group at the kingdom and species level, which is studied by the calculation of JSD distance/Bray Curtis distance from the abundance matrix (Fig. 1g–i).

**Figure 2:**
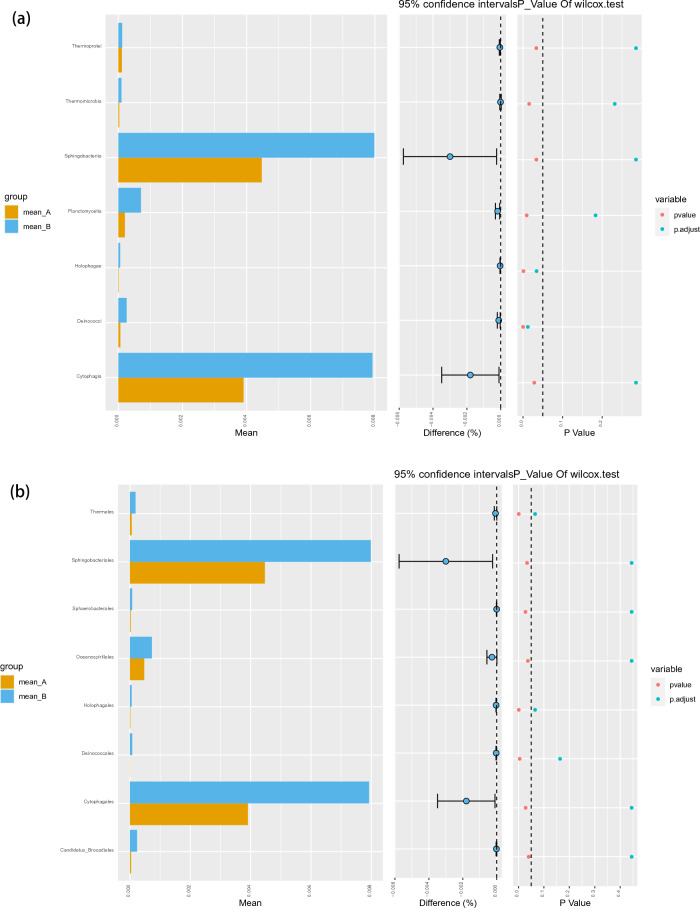

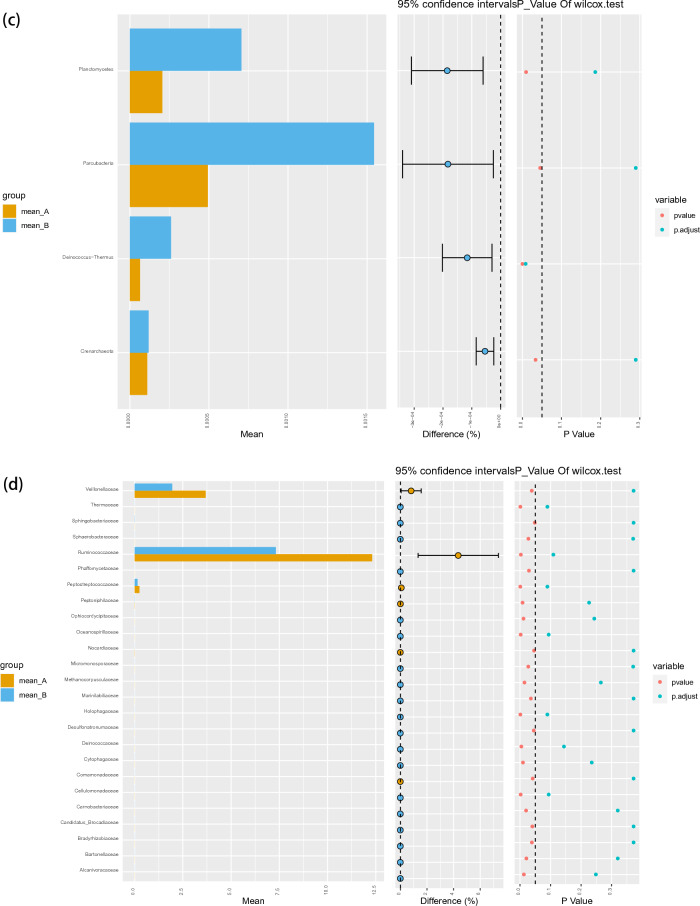

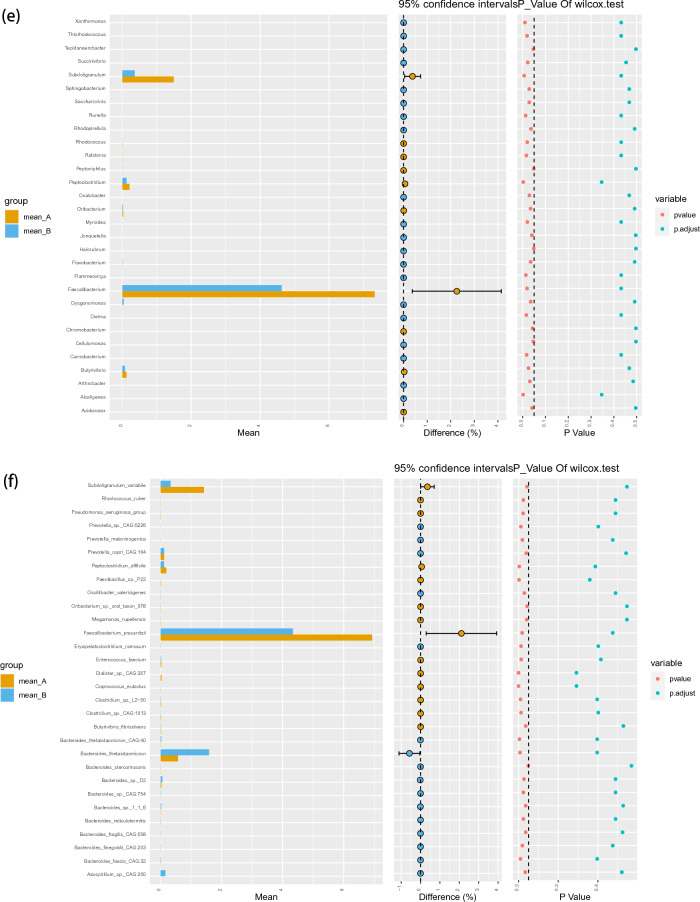
Abundances of taxa in ASD and TD groups. The mean relative abundances of taxa at class (**a**), order (**b**), phylum (**c**), family (**d**), genus (**e**) and species (**f**) levels in the ASD and TD groups. Yellow and blue bars indicate the mean relative abundances of taxa in the ASD and TD groups. *ASD* autism spectrum disorder, *TD* typically developing.

At the class level, *Sphingobacteriia* constituted the most abundant class in both the ASD and TD groups, but with no significant difference between the two groups (0.45 vs. 0.80%, P_FDR_ = 0.29). *Deinococci* (0.01 vs. 0.03%, P_FDR_ = 0.012) and *Holophagae* (0.00 vs. 0.01%, P_FDR_ = 0.034) were significantly lower in children with ASD compared with TD (Fig. 2a). At the order level, *Sphingobacteriales* constituted the most abundant class in both the ASD and TD groups, but with no significant difference between the two groups (0.09 vs. 0.08%, P_FDR_ = 0.95) (Fig. 2b). At the phylum level, *Parcubacteria* constituted the most abundant class in both the ASD and TD groups, but with no significant difference between the two groups (0.05 vs. 0.15%, P_FDR_ = 0.29). *Deinococcus*-Thermus (0.01 vs. 0.03%, P_FDR_ = 0.008) was significantly lower in children with ASD compared with TD (Fig. 2c).

**Figure 3:**
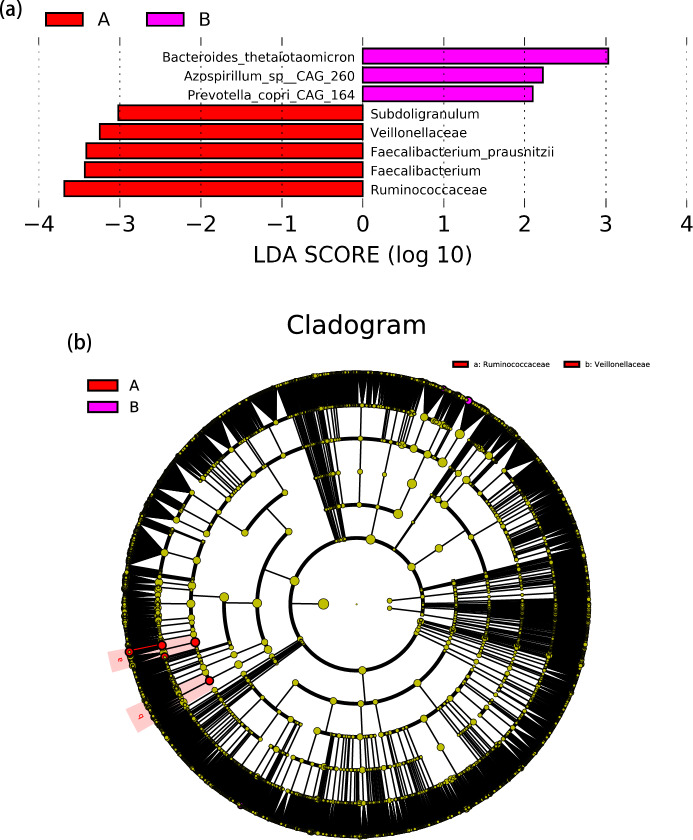
LEfSe analysis between ASD and TD groups. (**a**) LDA scores for the bacterial taxa differentially abundant between TD and ASD (LDA > 2.0). Red bars indicate taxa were enrichment in ASD, and rose red bars indicate taxa were enrichment in TD. (**b**) Cladograms generated by LEfSe indicating differences in the bacterial taxa between TD and ASD. Red bars indicate taxa were enrichment in ASD, rose red bars indicate taxa were enrichment in TD.

The linear discriminant analysis (LDA) distribution diagram analysis (LAD score > 2.0) showed a significantly higher relative abundance of the *Subdoligranulum*, *Faecalibacterium_praushitzii*, and *Faecalibacterium* genus and a significantly lower abundance of *Bacteroides_thetaiotaomicron*, *Azospirillum_sp_CAG_260* and *Prevotella_copri_CAG_164* taxa in the ASD group compared with the TD group. Similarly, a significantly higher relative abundance of *Veillonellaceae* and *Rumminococcaceae* family was observed in children with ASD than it was in TD children (*P* < 0.05) (Fig. 3).

**Figure 4:**
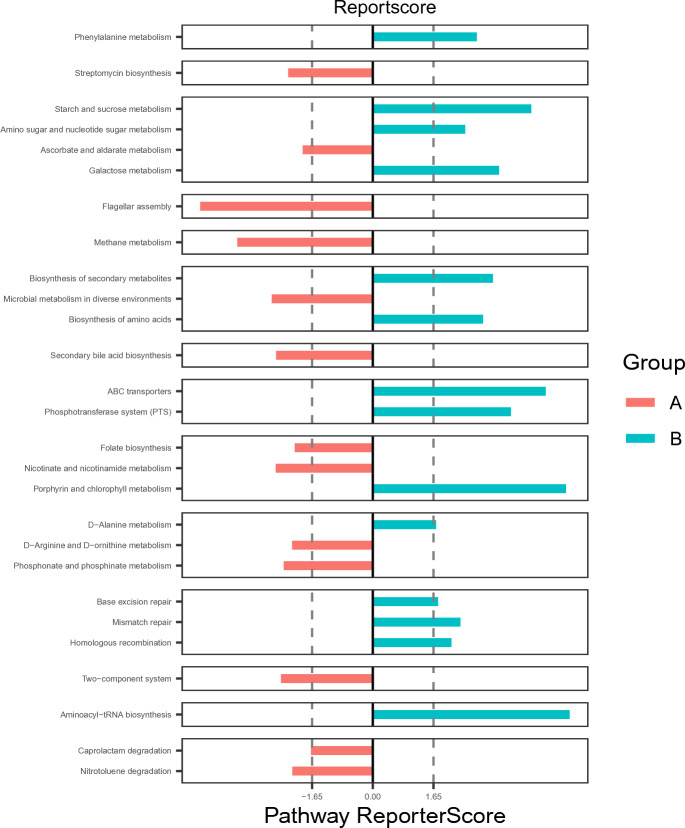
KEGG pathway differentially enriched in TD and ASD according to level 2. X-axis shows reporter score values, small blocks on Y-axis shows pathways. Differences are significant when bars exceed dashed lines.

The overall functional structure of the ASD group was dominated by 13 functions, such as *Streptomycin biosynthesis*, *Ascorbate and aldarate metabolism*, *Flagellar assembly*, *Methane metabolism*, and *Microbial metabolism in diverse environments*, while the TD group was dominated by the other 14 functions. Functional differences between groups were significant (Fig. 4).

**Figure 5:**
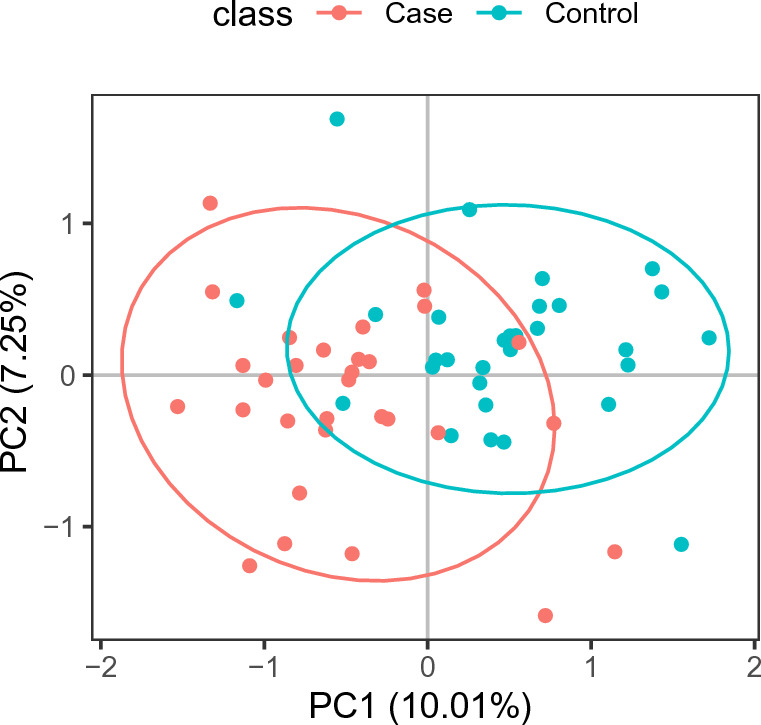
The clustering analyses of partial least-squares discriminant analysis (PLS-DA).

**Figure 6:**
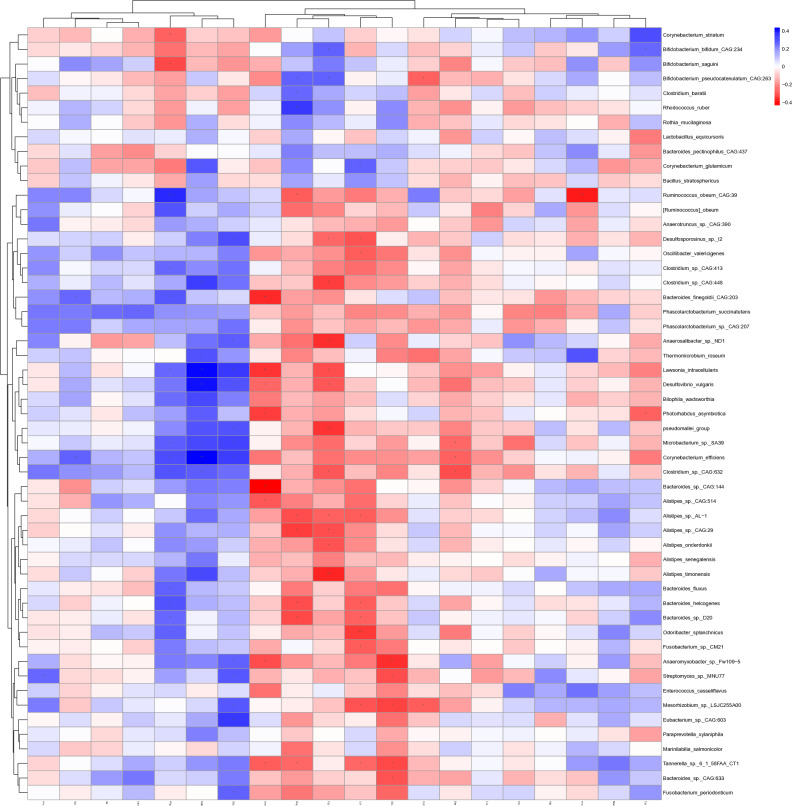
Correlations between species and metabolites. Metabolites ≥ 1.2-fold changes between TD and ASD, with *P* < 0.05, VIP ≥ 1. The correlation effect is indicated by a color gradient from red (negative correlation) to blue (positive correlation). * *P* < 0.05, ** *P* < 0.01.

Liquid chromatography-mass spectrometry (LC/MS) was used to examine the relationship between microbiota and metabolites. The blood samples from distinct groups were largely separated according to the Partial Least Squares Discriminant Analysis (PLS-DA) (Fig. 5), suggesting a dissimilar metabolic mode. To explore the potential relationships between the gut microbiome changes and metabolic products, a correlation matrix was generated using Spearman correlation (Fig. 6). The abundance of most species such as *Ruminococcus_obeum_CAG:39*, *Clostridium_sp._CAG:448*, *Lawsonia_intracellularis*, *Desulfovibrio_vulgaris*, *Corynebacterium_efficiens*, and *Eubacterium_sp._CAG:603*, were positively correlated with the level of Phenylalanine, Aspartic acid, and Glutamic Acid. *Bacteroides_finegoldii_CAG:203*, *Anaerosalibacter_sp._ND1*, *Lawsonia_intracellularis*, *pseudomallei_group*, *Bacteroides_sp._CAG:144*, *Alistipes_timonensis*, *Odoribacter_splanchnicus*, and *Anaeromyxobacter_sp._Fw109-5* were negatively correlated with the level of Asparagine, Arginine, Tryptophan, Citrulline, and Glutamine.

**Figure 7:**
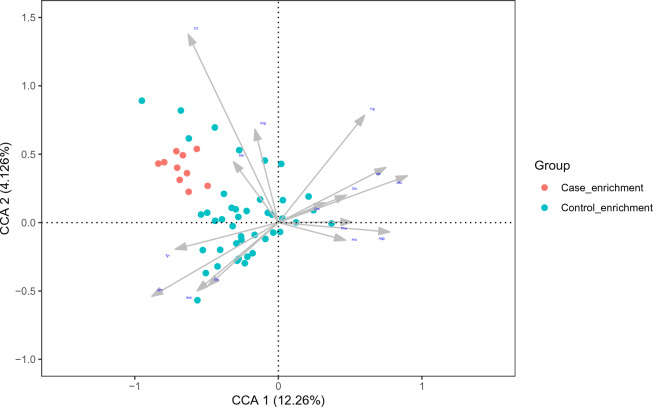
Canonical correspondence analysis between species and metabolites. The gray arrows represent the differential metabolites. The line length of the arrow represents the degree of correlation between a differentiated metabolite cluster and the overall microbial composition and community structure. The longer the line, the greater the correlation.

To explore which metabolites are closely associated with changes in differential species, a correlation matrix was generated using CCA (Fig. 7). Most amino acid metabolites are closely associated with changes in differential species such as Citrulline, Tryptophan, Leucine, Valine, Tyrosine, Asparagine, and Ornithine.

**Figure 8:**
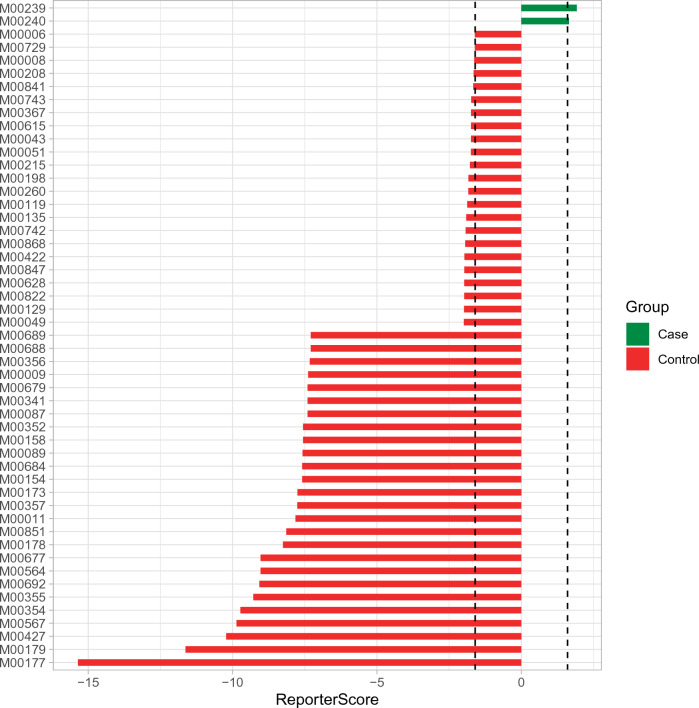
KEGG module differentially enriched in TD and ASD. X-axis shows reporter score values. The dotted line on the left represents reporter score = − 1.6, and the dotted line on the right represents reporter score = 1.6. Y-axis shows modules. Differences are significant when bars exceed dashed lines. M00239, Peptides/nickel transport system; M00240, Iron complex transport system; M00006, Pentose phosphate pathway, oxidative phase, glucose 6P=>ribulose 5P; M00729, Fluoroquinolone resistance, gyrase-protecting protein Qnr; M00008, Entner-Doudoroff pathway, glucose-6P=>glyceraldehyde-3P + pyruvate; M00208, Glycine betaine/proline transport system; M00841, Tetrahydrofolate biosynthesis, mediated by PTPS, GTP=>THF; M00743, Aminoglycoside resistance, protease HtpX; M00367, C10-C20 isoprenoid biosynthesis, non-plant eukaryotes; M00615, Nitrate assimilation; M00043, Thyroid hormone biosynthesis, tyrosine=>triiodothyronine/thyroxine; M00051, Uridine monophosphate biosynthesis, glutamine (+ PRPP) =>UMP; M00215, D-Xylose transport system; M00198, Putative sn-glycerol-phosphate transport system; M00260, DNA polymerase III complex, bacteria; M00119, Pantothenate biosynthesis, valine/L-aspartate=>pantothenate; M00135, GABA biosynthesis, eukaryotes, putrescine=>GABA; M00742, Aminoglycoside resistance, protease FtsH; M00868, Heme biosynthesis, animals and fungi, glycine=>heme; M00422, Acetyl-CoA pathway, CO2=>acetyl-CoA; M00847, Heme biosynthesis, archaea, siroheme=>heme; M00628, beta-Lactam resistance, AmpC system; M00822, Multidrug resistance, efflux pump MexMN-OprM; M00129, Ascorbate biosynthesis, animals, glucose-1P=>ascorbate; M00049, Adenine ribonucleotide biosynthesis, IMP=>ADP,ATP; M00688, MAPK (JNK) signaling; M00689, MAPK (p38) signaling; M00356, Methanogenesis, methanol=>methane; M00009, Citrate cycle (TCA cycle, Krebs cycle); M00087, beta-Oxidation; M00341, Proteasome, 19S regulatory particle (PA700); M00679, BMP signaling; M00158, F-type ATPase, eukaryotes; M00352, Spliceosome, U2-snRNP; M00089, Triacylglycerol biosynthesis; M00154, Cytochrome c oxidase; M00684, JAK-STAT signaling; M00173, Reductive citrate cycle (Arnon-Buchanan cycle); M00357, Methanogenesis, acetate=>methane; M00011, Citrate cycle, second carbon oxidation, 2-oxoglutarate=>oxaloacetate; M00851, Carbapenem resistance; M00178, Ribosome, bacteria; M00564, Helicobacter pylori pathogenicity signature, cagA pathogenicity island; M00677, Wnt signaling; M00692, Cell cycle-G1/S transition; M00355, Spliceosome, 35S U5-snRNP; M00354, Spliceosome, U4/U6.U5 tri-snRNP; M00567, Methanogenesis, CO2=>methane; M00427, Nuclear pore complex; M00179, Ribosome, archaea; M00177, Ribosome, eukaryotes.

*Peptides/nickel transport system* was the main metabolic pathway involved in the differential species in the ASD group, while the main metabolic pathway involved in the differential species in the normal group was *Eukaryotic ribosomes*. (Fig. 8).

**Figure 9:**
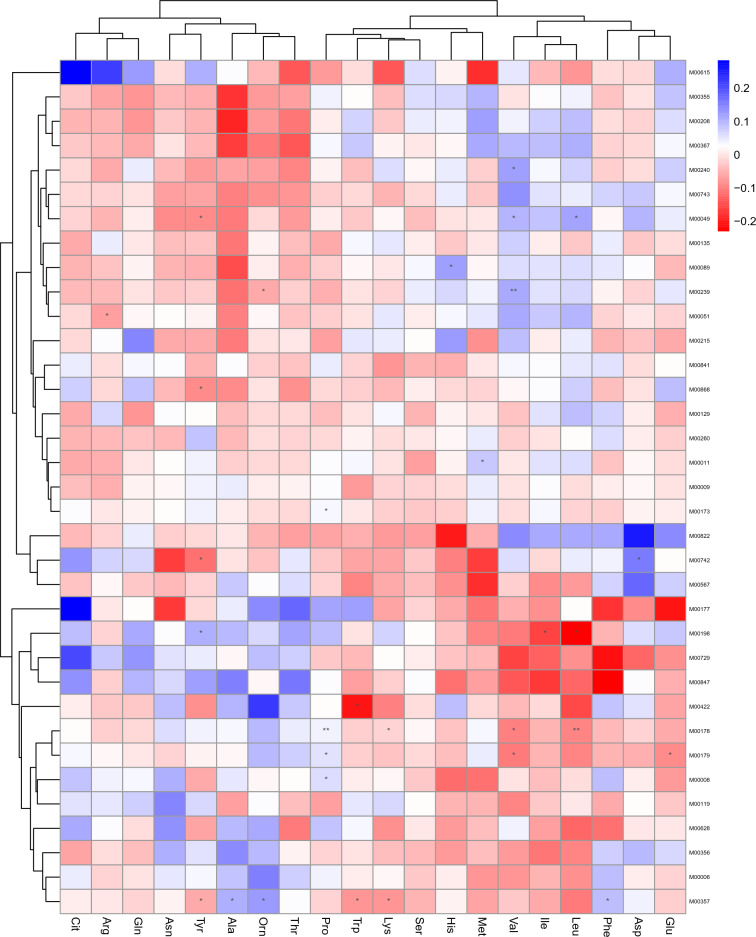
Heatmap shows the correlation of significantly enriched KEGG Module and differential metabolites. The correlation effect is indicated by a color gradient from red (negative correlation) to blue (positive correlation). * *P* < 0.05, ** *P* < 0.01.

The *peptide/nickel* transport system was positively correlated with valine levels (*p* < 0.01) and negatively correlated with ornithine levels (*p* < 0.05). (Fig. 9).

**Figure 10:**
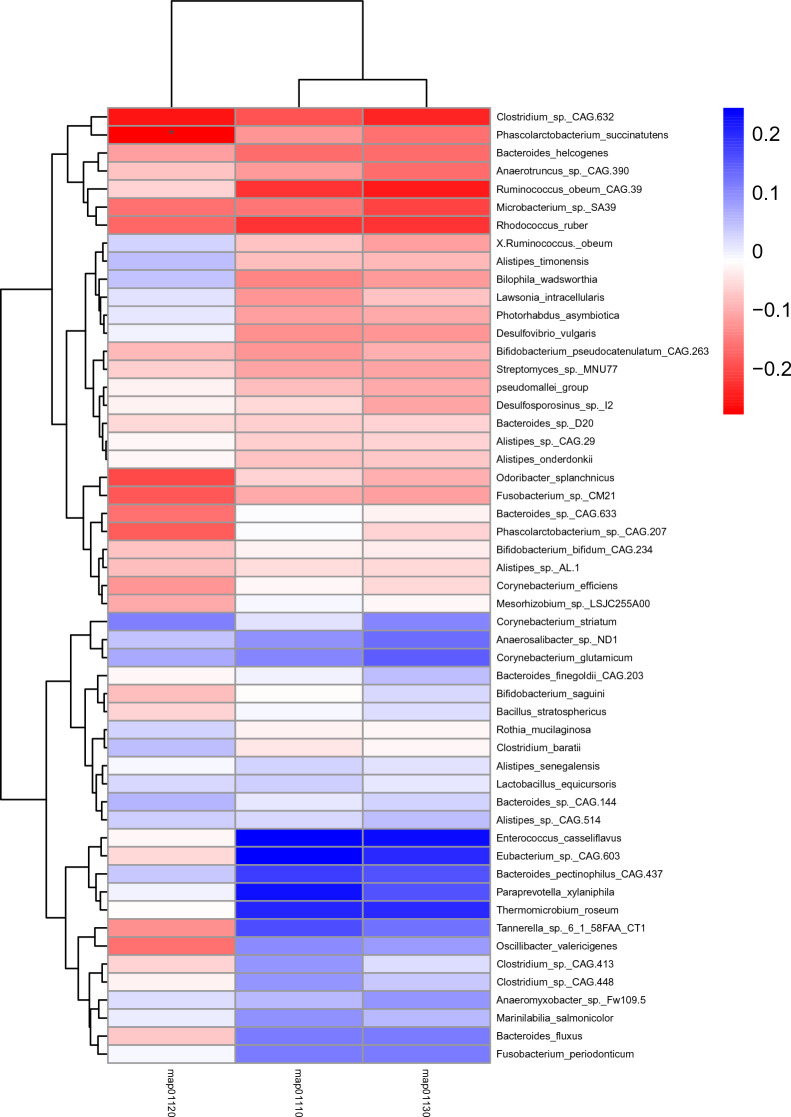
Heatmap shows the correlation of significantly enriched metabolite pathway and species. The correlation effect is indicated by a color gradient from red (negative correlation) to blue (positive correlation). * *P* < 0.05. map01120, Microbial metabolism in diverse environments; map01110, Biosynthesis of secondary metabolites; map01130, Biosynthesis of antibiotics.

The microbial metabolism in diverse environments was negatively correlated with *phascolarctobacterium succinatutens* (*p* < 0.05). (Fig. 10).

## Discussion

In our data, we found changes in the gut microbiota in children with ASD. We observed that higher richness in the gut microbiota in children with TD than in ASD, which is consistent with Ma's findings^33^. We also found the evenness of species was significantly higher in the ASD group than that in TD at the kingdom level, which had not been reported previously. Beta diversity analysis revealed different microbiome characteristics at kingdom and species levels between the two groups. This indicated that the microbial community structure of the ASD group had changed, a finding supported by previous studies from different countries^34,35^.

Our data analysis showed a significant reduction of *Deinococci* and *Holophagae* in the fecal samples of ASD subjects compared to the TD group at the class level, which had not been reported previously. The class *Holophagae* is a member of the phylum *Acidobacteria*, *Acidophilus* may be used as a probiotic to promote the growth of good bacteria in your body. *Acidophilus* may also help treat a variety of medical conditions such as digestive issues^36^. Comparing the microbial taxa at the phylum level between the ASD and TD groups, *Deinococcus*-Thermus was significantly lower in children with ASD in the present study. Features such as the accumulation of genes encoding cell cleaning systems that eliminate organic and inorganic cell toxic components are widespread among *Deinococcus* spp^37^.

The LDA distribution diagram analysis showed a significantly higher relative abundance of *Veillonellaceae* and *Rumminococcaceae* family in the ASD group compared with the TD group, a finding consistent with previous studies^38,39^. Compared with the TD group, the relative abundance of *Prevotella_copri_CAG_164* in the ASD group was identified as decreased significantly. This result was in agreement with previous studies^38,40^. *Prevotella copri* can utilize polysaccharides to produce succinic acid^41^, which has been reported to enhance the immune response of antigen-specific T cells by binding to the succinic acid receptor GPR91 on the surface of dendritic cells to protect host health^42^. Many ASD children display immune dysfunction^43^, their immune disorder may be related to the decrease of Prevotella bacteria in the intestine.

Among the thirteen functions of hypermetabolism in the ASD group, it was found that arginine metabolism is related to the cognitive development of the brain, while argininemia can affect the development of the brain^44^. It was also found that phenylalanine metabolism and amino acid biosynthesis are related to neurodegeneration, which suggests that gut microbiota may affect the central nervous system through this pathway^45,46^.

Most metabolites are closely associated with changes in different amino acids such as citrulline, tryptophan, leucine, valine, tyrosine, asparagine, and ornithine. Studies have shown that tryptophan and tyrosine levels in children with ASD are lower than in controls^47,48^. Leucine and valine are branched-chain amino acids (BCAA). Elevated BCAA will cause catabolic abnormalities, which may lead to mitochondrial dysfunction. Current studies have shown that most patients with ASD will have mitochondrial dysfunction, which is believed to be related to its etiology^49^. In addition, lack of expression of SLC7A5, an amino acid transporter on the blood–brain barrier, leads to a significant decrease in the level of branch chain amino acids, especially leucine and isoleucine, resulting in the occurrence of ASDs^50^. One study showed that ADOS-2 restricted and repetitive behavior scores were positively correlated with citrulline levels in the ASD group, and the amino acid profile in the ASD group showed reduced levels of ornithine^51^. Elevated levels of asparagine are the distinct characteristics of the plasma amino acid profile of autistic children^52^.

*Peptides/nickel transport system* was the main metabolic pathway involved in the differential species in the ASD group, while the main metabolic pathway involved in the differential species in the normal group was *Eukaryotic ribosomes.* The nickel transport system is the specific transport system of Helicobacter pylori^53^. Helicobacter pylori infection may increase the risk of neurodegenerative disease^54^. Correlation analysis of differential metabolites and the KEGG Module showed that the *peptide/nickel* transport system was positively correlated with valine levels and negatively correlated with ornithine levels. It suggests that decreased ornithine levels and elevated valine levels may increase the risk of ASD through a metabolic pathway known as the nickel transport system. Ribosomes are protein processing machines, responsible for decoding the codon information encoded by mRNA into the corresponding amino acid sequence of proteins. Eukaryotic ribosomes are more accurate at decoding mRNA than bacterial ribosomes, and in humans, changes in decoding fidelity have been linked to diseases such as neurodegenerative diseases and represent potential therapeutic intervention targets^55^.

Spearman correlation analysis was conducted between the significantly enriched metabolites pathway and species, and the results showed that the microbial metabolism in diverse environments was negatively correlated with *P. succinatutens*, which had not been reported previously. It may be due to the effect of microbial metabolism in diverse environments on the physiological process of *phascolarctobacterium*. *Phascolarctobacterium* was a component of a stable microbiota community in children with ASD^56^. The genus *Phascolarctobacterium* was much more abundant in ASD children than in the neurotypical participants^57^. The discovery of this correlation could provide clues for further study of the physiological processes of *P. succinatutens*.

## Conclusions

In summary, this study revealed that ASD patients showed gut dysbiosis at both phyla and class levels. The altered species associated with the alteration of ASD metabolites were identified. By analyzing the interactions between the gut microbiota and metabolites, we can better understand the mechanisms of altered social behavior in patients with ASD and provide clues to reveal whether such changes are related to the gut microbiota. Abnormal metabolites may have important implications in the pathogenesis of ASD. Because it is unclear whether there is a causal relationship between gut microbiome changes and ASD, large-scale prospective studies are still needed to validate our results. However, this study provides insights into the relationship between the fecal microbiome and metabolites in patients with ASD, which also provides a possible model for ASD interventions targeting specific microbiota related to amino acid metabolism.

## Data Availability

The datasets generated and/or analyzed during the current study are not publicly available due to institutional policy but are available from the corresponding author upon reasonable request.
